# Core transcriptional regulatory circuitries in cancer

**DOI:** 10.1038/s41388-020-01459-w

**Published:** 2020-09-17

**Authors:** Ye Chen, Liang Xu, Ruby Yu-Tong Lin, Markus Müschen, H. Phillip Koeffler

**Affiliations:** 1 Cancer Science Institute of Singapore, National University of Singapore, Singapore, 117599 Singapore; 2 Department of Systems Biology, City of Hope Comprehensive Cancer Center, Monrovia, CA 91016 USA; 3 College of Life Sciences, Zhejiang University, Hangzhou, 310058 China; 4 Department of Medicine, Cedars-Sinai Medical Center, Los Angeles, CA 90048 USA; 5 National University Cancer Institute, National University Hospital, Singapore, 119074 Singapore

**Keywords:** Cancer genetics, Transcription

## Abstract

Transcription factors (TFs) coordinate the on-and-off states of gene expression typically in a combinatorial fashion. Studies from embryonic stem cells and other cell types have revealed that a clique of self-regulated core TFs control cell identity and cell state. These core TFs form interconnected feed-forward transcriptional loops to establish and reinforce the cell-type-specific gene-expression program; the ensemble of core TFs and their regulatory loops constitutes core transcriptional regulatory circuitry (CRC). Here, we summarize recent progress in computational reconstitution and biologic exploration of CRCs across various human malignancies, and consolidate the strategy and methodology for CRC discovery. We also discuss the genetic basis and therapeutic vulnerability of CRC, and highlight new frontiers and future efforts for the study of CRC in cancer. Knowledge of CRC in cancer is fundamental to understanding cancer-specific transcriptional addiction, and should provide important insight to both pathobiology and therapeutics.

## Introduction

**Fig. 1:**
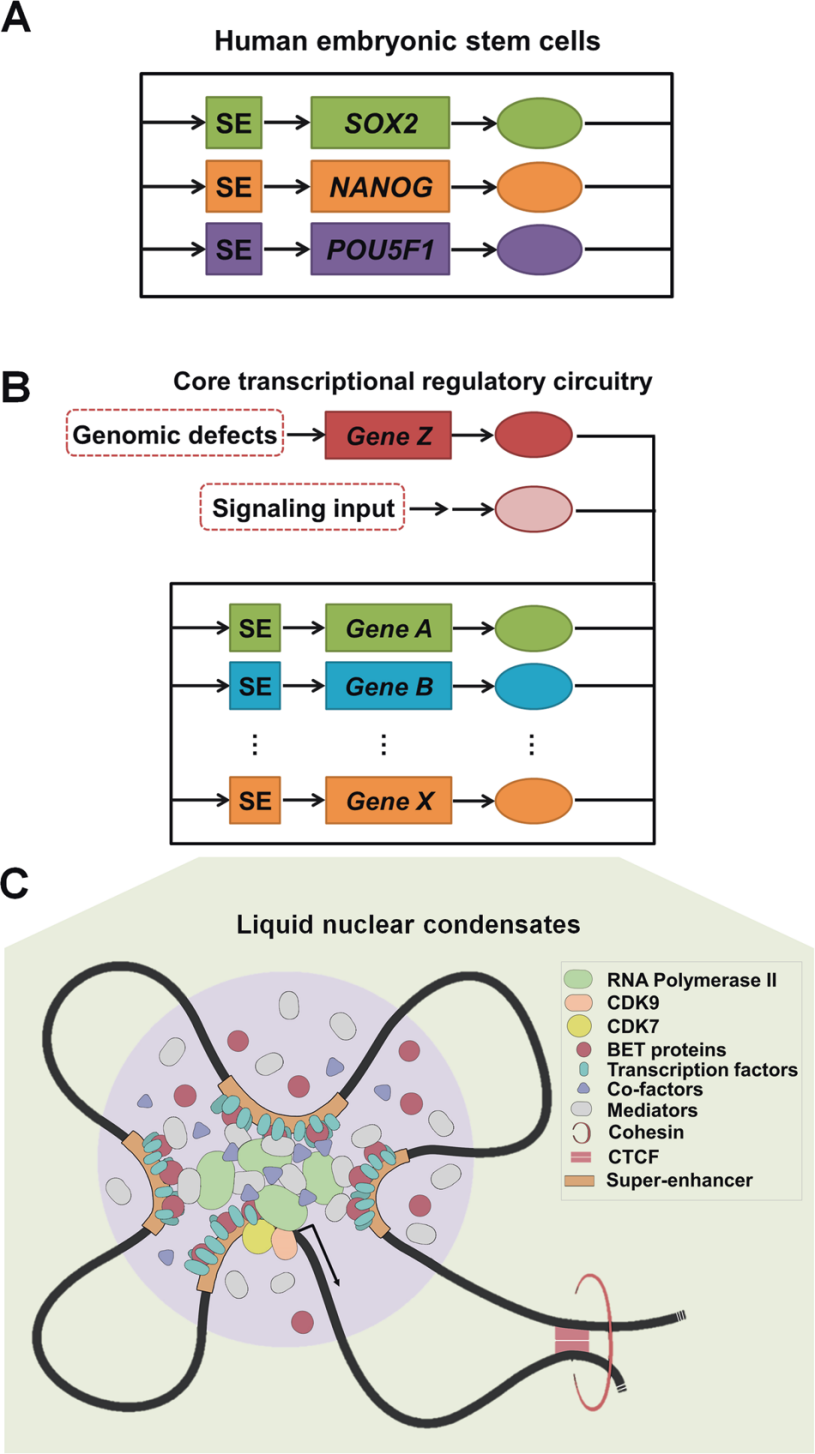
Genetic and molecular mechanisms of core transcriptional regulatory circuitry. **a** The first model of core transcriptional regulatory circuitry in human embryonic stem cells. Gene loci and super enhancers are depicted as rectangles. **b** Convergence of genomic defect and oncogenic signaling dysregulation on feed-forward core transcriptional circuit in human cancer. **c** A model of phase-separated liquid condensates in regulating super enhancer (SE) driven gene expression. SE domains enrich asymmetric loading of core TFs, BET bromodomain proteins and additional chromatin regulatory factors/co-factors (e.g., p300 and HDACs). Regionally concentrated BET proteins, mediators, and RNA polymerase II are capable to form liquid condensates to favor productive transcription of SE targets within a topologically associating domain.

Transcriptional regulation is one of the fundamental molecular processes occurring in a cell. Sequence-specific DNA-binding proteins, also known as transcription factors (TFs), orchestrate gene-expression patterns in various cell types and growth conditions [1]. Although thousands of TFs have been identified, only a limited cohort of master TFs controls the core transcriptional programs governing cell identity [2–4]. Master TFs are highly expressed in a given cell type. Master TFs bind to the majority of cell-type-specific enhancers and dictate expression of cell-type-specific genes. Till now, one critical goal in biology remains to understand the composition and hierarchy of transcriptional regulatory network in each specified cell type/lineage. One of the best-studied models is embryonic stem cells (ESCs). In ESCs, three master TFs (pluripotency factors) NANOG, POU5F1/OCT4 and SOX2 form interconnected feed-forward transcriptional loops to maintain gene-expression program associated with ESC identity (Fig. 1a) [5, 6]. Such a pluripotent transcriptional regulatory network nurtured the model/concept of core transcriptional regulatory circuitry (CRC) in ESCs [7]. Apart from core TFs, additional layers of core regulators have been discovered and integrated into the pluripotent transcription circuitry including external signaling pathways [8], chromatin/histone modifiers (Polycomb group proteins [9], histone acetyltransferase MOF [10], and WDR5 [11]), basal transcription machinery TF IID complex [12], histone modifications (H3K56ac [13]), noncoding RNAs (microRNAs [14] and lncRNAs [15]), and transposable elements [16]. Insights from ESC-associated CRC have guided subsequent exploration of similar core feed-forward transcriptional networks during lineage specification, development, and tumorigenesis [17–23]. These studies suggest widespread existence and critical function of CRCs in both physiological and pathological conditions.

This review mainly focuses on the progress of CRC studies in human cancers, diseases of uncontrolled cell growth. We have learned from genomic sequencing projects that cancer is driven by genetic alterations [24]. Oncogenic genetic abnormalities invariably reprogram transcriptome in order to establish and maintain cancerous identity/state [25]. As featured by defective terminal differentiation, cancer cells are often locked into a growth state resembling either stem/progenitor cells or cells in a certain developmental stage. A growing body of evidence shows that dysregulated transcriptional programs in cancers are dominated by a clique of interconnected master TFs [21, 26–30]. These master regulators form feed-forward autoregulatory loops, and function in a combinatorial way to enhance expression of cancer-promoting genes. Aberrations in cancer genome, signaling transduction, and epigenetic regulation (e.g., super enhancer (SE)) activate cancer-specific expression of master TFs, which frequently hijack lineage-specific TFs, pioneer factors, epigenetic readers, mediators, and chromatin-regulating machineries to reshape epigenome [4, 21, 29, 31–35]. Cancer-specific activation of core TFs and subsequent rewiring of lineage-associated CRC components establish the genetic basis of oncogenic CRCs (Fig. 1b). Phase-separated condensation of core TFs, BRD4, mediators, and RNA polymerase II emerges as a biophysical mechanism to ensure high local availability of chromatin regulators, and transcription machineries for productive oncogenic transcription (Fig. 1c) [36–38].

Elucidating the core transcriptional regulatory programs will provide better understanding of molecular carcinogenesis. Lineage-specific components in CRC can inform on both cell-of-origin in cancer and selective oncogenic dependencies. Moreover, disruption of CRC in cancer cells by either genetic approach or pharmacological inhibition greatly impairs their malignant characteristics and tumorigenicity. Thus, CRC represents a mechanism of oncogenic addiction and a potential target for novel therapeutic interventions in cancer. Here, we summarize recent efforts to identify, characterize, and target CRCs across various human cancers, and highlight key insights that have emerged from these seminal studies.

## Strategy and methodology for CRC identification

Inspired from ESC studies, self-regulation and interconnection are two important mechanisms to stabilize TF network. Key features of CRC include (1) self-regulated expression of each core TF, (2) direct regulation among core factors, and (3) feed-forward transcriptional control. Hence, identification of CRC is highly dependent on mapping of TF binding sites and biological verification of cross regulation. Genomic occupancy analysis of candidate core TFs enables systematic annotation of their direct targets. Indeed, initial modeling of CRCs in ESCs [5], hepatocytes [18], and T-cell acute lymphoblastic leukemia (T-ALL) [21] was based on genome-wide discovery of TF-DNA interactions via chromatin immunoprecipitation (ChIP) followed by either microarray hybridization or high-throughput sequencing (ChIP-seq). Typically, core TFs occupy their own promoters/enhancers. Meanwhile, core TFs often bind in close proximity to cis-regulatory elements of their target genes, producing a “co-occupancy” pattern of genomic binding. Potential biochemical mechanism of this “co-occupancy” pattern is associated with either direct protein–protein interactions (e.g., SOX2-OCT4 heterodimer) or co-existence of core TFs within multi-subunit protein complexes. The mutual co-occupancy of core TFs within their own cis-regulatory elements constitutes an interconnected autoregulatory loop, while co-loading of TFs across the vast majority of downstream target genes implicates their substantial co-operation in gene regulation.

**Fig. 2:**
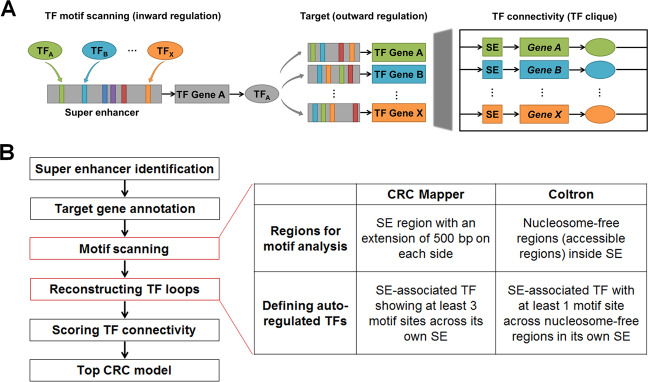
Modeling of core transcriptional regulatory circuitry. **a** Schematic of enhancer-centric reconstruction of core transcriptional regulatory circuitry. **b** Flow chart showing the key steps of CRC reconstruction by CRC Mapper and Coltron. Both methods model CRC based on TF connectivity among SE regions, while they differ in several parameters during steps of motif scanning and reconstructing TF loops.

To ensure feed-forward regulation within CRC, core TFs commonly bind to cis-regulatory elements and open chromatin regions, including promoters, enhancers, DNase I hypersensitive sites, and SEs [4]. Since TF motifs are overrepresented in genomic regions occupied by respective TFs, systematic identification of TF motifs across the cis-regulatory elements of a given sample will provide raw materials to reconstruct a regulatory network. SEs have been characterized as constituent enhancers, which are bound densely by multiple TFs [4, 31]. Since SEs are frequently engaged in expression of lineage-specific and/or disease-promoting genes including core TFs, SE-driven TFs serve as promising candidates for core TFs in CRC. Based on the principles of “self-regulation” and “interconnection,” several computational programs have been developed to wire potential regulatory nodes and to model CRC. Saint-André et al. [23] developed a “CRC Mapper” program to reconstruct human CRC models of SE-associated TFs (Fig. 2a). This approach utilized FIMO software [39] package from the MEME suite [40] to scan TF motifs inside SE regions (with 500-bp extension of each SE boundary), and then retrieved both auto-regulated TFs and all possible fully interconnected regulatory circuitries. Top-scoring circuitry of which TFs exhibited the highest frequency of occurrence across all predicted circuitries was designated as CRC in a given sample. “CRC Mapper” has demonstrated excellent performance to recapitulate experimentally verified CRCs in ESCs and T-ALL. So far, this program has been implemented to build CRC models in a large variety of samples [19, 41, 42]. Remarkably, dbCoRC (http://dbcorc.cam-su.org), a comprehensive online database of “CRC Mapper”-inferred circuitries in over 230 human/murine samples has been developed and is freely accessible, representing a valuable resource to explore transcriptional regulatory networks [41]. Using similar principles of motif-based TF connectivity for network construction, Lin et al. [26] developed a “Coltron” python package (https://pypi.org/project/coltron), which quantified degree of inward (IN) and outward (OUT) regulation of SE-regulated TFs (NODEs) across putative nucleosome-free regions (NFRs) of their constituent enhancers. Based on motif analysis, IN-degree for a given SE-regulated TF (NODE) was defined as the number of SE-regulated TFs bound to its proximal/assigned SE; OUT-degree was calculated as the number of TF-associated SEs bound by a given SE-regulated TF. Total degree (IN + OUT) of each NODE can be applied to rank the connectivity of TFs. CRC was defined as the top-ranked autoregulatory TF network (CLIQUE), which showed highest average enrichment of core TFs among all CLIQUEs [27]. The capability of “Coltron” to predict motif-based TF binding is enhanced, as it scans TF motifs inside NFRs instead of whole SE domain (Fig. 2b) [43]. Further, either incorporation of experimentally derived TF footprints data (DNase I hypersensitive sites) or accessible regions from ATAC-seq (Assay for Transposase-Accessible Chromatin using sequencing) can improve the prediction of TF binding sites [2, 27, 44–46]. In addition to genomic occupancy and TF connectivity, regulatory readout of core TFs and their functional impact on cell behaviors are other fundamental aspects for CRC. Since core TFs with feed-forward transcriptional regulation often show positively correlated expression, co-expression analysis of TFs among cancer type/subtype of interest can be employed to shortlist candidate core TFs. Furthermore, genetic manipulation assays represent the “gold standard” to validate both regulatory circuitry among core TFs and functional importance of TFs in maintaining cell identity. Depletion of individual core TFs will reduce expression of other members within CRC, and subsequently impair cellular phenotypes (e.g., proliferation and differentiation). Systematic analysis of putative downstream targets will also help to identify important mediators/executors in regulating cell identity.

### Experimentally verified CRCs and their implications in cancer pathobiology and therapeutics

To gain insight into the roles of CRCs in human cancers, we next summarize current knowledge of biologically verified CRCs in human cancers.

#### T-cell acute lymphoblastic leukemia (T-ALL)

**Fig. 3:**
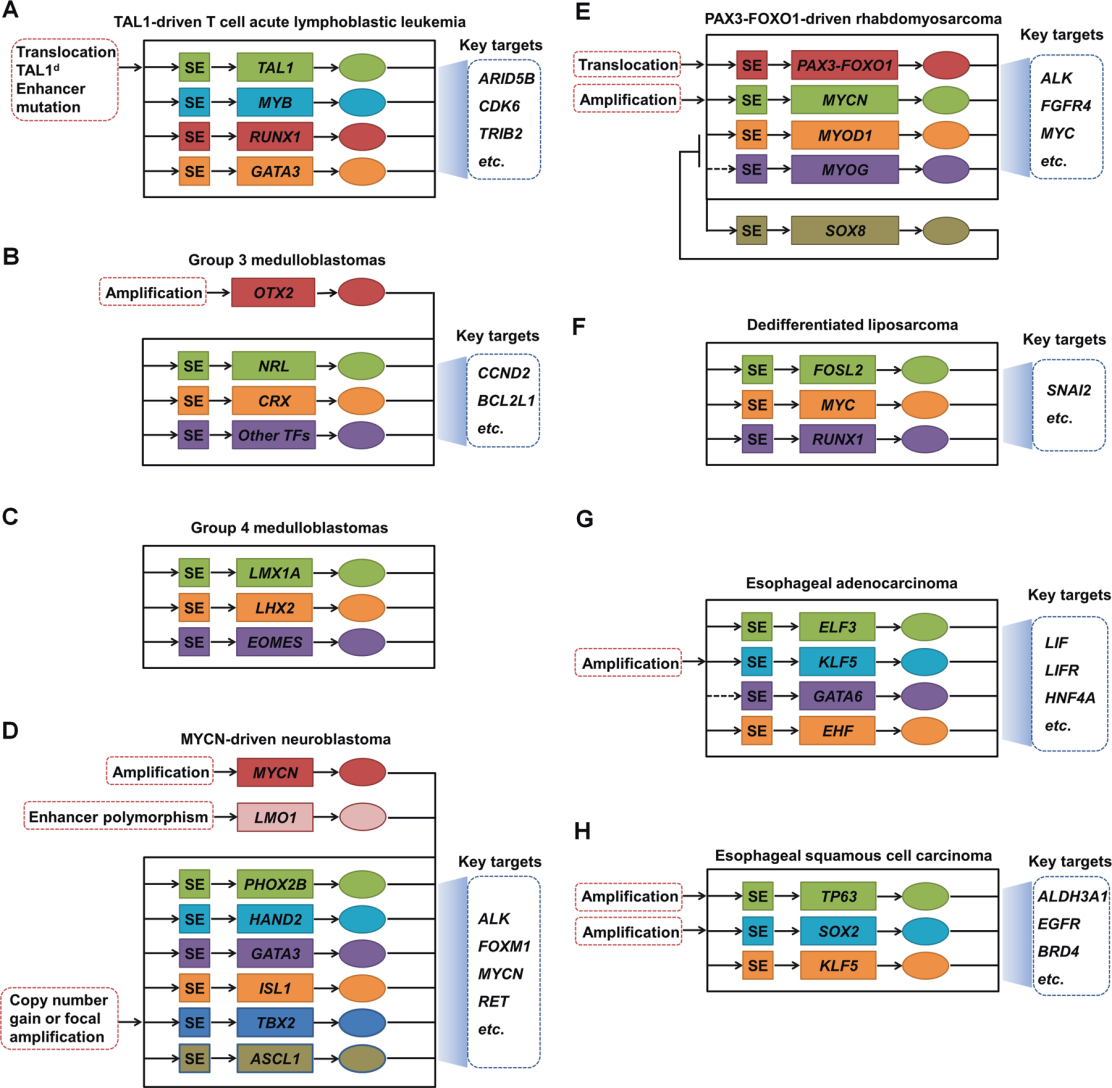
Experimentally verified core transcriptional regulatory circuitries in human cancer. Diagrams showing the core regulatory circuitries and key downstream targets from extended network in **a** TAL1-positive T-cell acute lymphoblastic leukemia, **b** group 3 medulloblastoma, **c** group 4 medulloblastoma, **d** MYCN-driven neuroblastoma (sympathoadrenal subtype), **e** fusion-positive rhabdomyosarcoma, **f** dedifferentiated liposarcoma, **g** esophageal adenocarcinoma and **h** esophageal squamous cell carcinoma. Genomic alterations that contribute to the aberrant expression of core TFs in each disease are indicated in the red dashed boxes. Representative downstream targets of core regulatory circuitries are listed in blue dashed boxes. Gene loci and super enhancers are depicted as rectangles.

T-ALL is a disease with cancerous proliferation of immature thymocytes. Genetic lesions affecting an oncogenic TF TAL1/SCL are frequently observed in human T-ALL cells [47]. Either chromosomal translocation involving T-cell receptor loci or intrachromosomal deletion of an 80-kb DNA fragment in 1p33 involving STIL (SIL-TAL1 deletion or TAL1^d^) has been shown to contribute to the overexpression of TAL1 [47]. TAL1 activation blocks T-cell differentiation, reactivates stem cell genes, and leads to malignant transformation [48]. In TAL1-positive T-ALL (e.g., Jurkat and CCRF-CEM cells), Sanda et al. identified a positive feed-forward regulatory circuitry among TAL1, RUNX1, and GATA3 based on ChIP-seq and expressional analyses [21]. TAL1 transcriptional complex included HEB, E2A, LMO1/2, and MYB; MYB was further established as an additional core TF to extend the aforementioned core regulatory loop in T-ALL [49] (Fig. 3a). Remarkably, Mansour et al. reported a mechanism of monoallelic TAL1 activation involving somatic mutation of an enhancer element at 7.5-kb upstream of the transcription start site of TAL1 [49]. These mutations introduced de novo MYB binding site to the TAL1 SE, facilitating the subsequent recruitment of MYB, histone acetyltransferase CBP, and additional components of TAL1 complex. CRISPR/Cas9-mediated deletion of TAL1 enhancer mutation profoundly impaired both MYB binding and H3K27ac signals in this SE. Of note, TAL1, RUNX1, GATA3, and MYB bound to the respective enhancers or SE regions of each other, as well as those of their own. They also co-occupied the regulatory elements of many downstream targets, such as the leukemogenic TRIB2, ARID5B, CDK6, and lncRNAs [50–52]. Among these targets, ARID5B was further characterized as a collaborating oncogenic factor to maintain expression of core TFs and participate in the transcriptional program of T-ALL [51]. Functional studies demonstrated that depletion of either TAL1 or other components of the circuitry not only disrupted the expression of all core TFs, but also attenuated survival of TAL1-positive T-ALL cells. In addition, RUNX1 has been reported to be required for the growth of both TAL1-induced murine T-ALL cells and human T-ALL cells [53]. Given the prevalent alterations of TAL1 expression in T-ALL, activation of TAL1 and subsequent establishment of T-ALL-associated CRC are likely the primary events during malignant transformation of TAL1-positive cases. Either selective degradation of bromodomain and extra terminal (BET) proteins or targeted inhibition of kinases of RNA polymerase II carboxyl-terminal domain (e.g., CDK7 and CDK9) effectively collapsed the core regulatory circuitries in T-ALL cells, hence exhibiting potent antineoplastic activities [54–56].

#### Medulloblastoma

Medulloblastoma is the most common type of pediatric brain tumor, which can be classified into four molecular subtypes: WNT, SHH, Group 3, and Group 4 [57]. Lin et al. charted the active enhancer landscape of 28 primary medulloblastoma specimens based on simultaneous profiling of DNA methylome, transcriptome, and chromatin loading of BRD4 and H3K27ac [26]. In this study, “Coltron” was developed for computational reconstruction of subtype-specific TF regulatory networks. This analysis of SE-regulated TFs identified cliques of TFs with a high likelihood of motif co-occurrence, interconnectivity, and protein–protein interaction. A CRC blueprint was generated for each subgroup, providing valuable insights to heterogeneous identities of medulloblastomas. WNT-subtype medulloblastomas may be driven by core TFs including LEF1, MAF, RUNX2, and EMX2. SHH subtype potentially enrolled OTX1, BARHL2, MAFF, and GLI2 as core TFs. Group 3 tumors were initially predicted to enrich CRX, HLX, LHX2, and OTX2, yet the top-ranked CRC model was revised to include OTX2, NRL, and CRX (Fig. 3b) after re-analysis of TF-enhancer interaction networks using an updated TF motif database (allocation of MAF motifs to NRL motifs) [58]. In support of this, OTX2 is frequently amplified and overexpressed in this group [59, 60]. OTX2 has been shown as a pathogenic TF that shapes the chromatin landscape of Group 3 medulloblastomas [61]. Further, OTX2 was recruited to SE regions of NRL and CRX. OTX2 expression also showed a strong positive correlation with both NRL and CRX expression in this subtype. Meanwhile, NRL and CRX were inferred to occupy ~2/3 of all SE-associated genes in Group 3 [58]. Functionally, these two core TFs cooperated to regulate a photoreceptor transcriptional program and expression of several oncogenes (e.g., CCND2 and BCL2L1), suggesting identity of photoreceptor lineage for Group 3 medulloblastomas. Group 4 medulloblastomas relied on the feed-forward co-expression of LMX1A, LHX2, and EOMES (Fig. 3c) [26]. Group 4-specific regulatory circuitry was partially verified by ChIP-seq analysis of LMX1A and LHX2. Notably, Group 4 CRC also resembled an expressional pattern of progenitors in cerebellar upper rhombic lip, suggesting a potential cell-of-origin for these tumors. Collectively, these medulloblastoma studies exemplify insightful applications of CRC reconstruction in understanding of tumor cell-of-origin and pathobiology.

#### Neuroblastoma

As a cancer derived from multipotent neural crest cells (NCCs) of the peripheral sympathetic nervous system, neuroblastoma represents one of the most common pediatric malignancies. Boeva and colleagues applied “Coltron” to infer CRC models from SE landscapes of human neuroblastoma cell lines, patient-derived xenografts (PDXs), and primary NCC lines [28]. They reported three types of identity among these samples. Group 1 samples comprised the majority of neuroblastoma cell lines and PDXs, and engaged SE-associated master TFs (e.g., PHOX2B, HAND2 and GATA3) to control sympathetic adrenergic cell identity. This module of core TFs showed strong co-expression and constituted a conserved CRC in both group 1 neuroblastoma cell lines and PDXs. In addition to the physical interaction between PHOX2B and GATA3, all three TFs co-localized in the regulatory sequences of their own loci, as well as many other prominent driver genes including MYCN and ALK. Group 2 samples resembled the SE pattern of NCCs, which were driven by a CRC module containing AP-1 TFs, e.g., FOSL1 and FOSL2. The third group of neuroblastomas was characterized by a mixture of both Groups 1 and 2 modules; indeed, most primary neuroblastoma specimens exhibited mixed expression of TFs of both modules. In line with the observation that most MYCN-amplified neuroblastoma cells enriched the adrenergic TF module, functional genomics studies with CRISPR-Cas9 screen identified MYCN, PHOX2B, HAND2, GATA3, and additional SE-regulated TFs including ISL1 and TBX2 as key dependency genes showing selective requirement for proliferation and survival of MYCN-amplified neuroblastoma cells [32]. Compound depletion of MYCN and TBX2 exerted a greater deleterious impact on both cell growth and downstream FOXM1/E2F network [62]. Notably, MYCN, PHOX2B, HAND2, GATA3, ISL1, and TBX2 demonstrated clustered binding across open chromatin regions (as “epicenters”) of their own genes and interdependent expression. ASCL1 and an auxiliary adapter protein (LMO1) further expanded this adrenergic transcriptional regulatory circuitry by co-binding to target regions (Fig. 3d) [63]. Both ASCL1 and LMO1 maintained proliferative potential of neuroblastoma cells. ASCL1 overexpression also suppressed pathways involved in the development of sympathetic nervous system and noradrenergic lineage commitment.

Remarkably, MYCN and TBX2 are frequently targeted by somatic genomic alterations in high-risk neuroblastoma samples. Genomic amplification of MYCN rendered its function as an exceptional CRC member whose overexpression surpassed the regulatory impact from many other core TFs [32]. In contrast, in case of cells with single copy of MYCN (e.g., CLB-GA), MYCN expression was dependent on TBX2 [62]. Segmental gains and rare focal amplification of chromosome 17q coincided with elevated expression of TBX2 [62]. Germline single nucleotide polymorphism (rs2168101 G>T) within intron 1 of LMO1 has also been associated with predisposition to neuroblastoma [64]. This risk allele increased LMO1 expression by creating a GATA3 binding site within its SE element. Hence, both somatic and germline genomic changes contribute to neuroblastoma development via aberrant activation of adrenergic transcriptional regulatory module.

Several attempts to disrupt the adrenergic transcriptional regulatory circuitry in neuroblastoma have been made, including combined treatment of CDK7 inhibitor (THZ1) with either BET protein inhibitor (JQ1) or HDAC inhibitor (Panobinostat) [32, 62]. Combination of THZ1 and JQ1 demonstrated synergistic repressive effects on both cell viability and CRC gene expression. Since plasticity of CRCs in neuroblastomas has been observed during chemotherapeutic treatment [28], incorporation of CRC-disrupting agents into current therapeutic scheme may provide additional benefit to neuroblastoma patients.

#### Rhabdomyosarcoma (RMS)

RMS is an aggressive soft-tissue cancer that arises from skeletal muscle precursors. Fusion-positive rhabdomyosarcoma (FP-RMS) is defined by translocations involving chimeric TFs either PAX3-FOXO1 (P3F) or PAX7-FOXO1 [65]. Amplification of MYCN is also frequently detected in FP-RMS [66]. Four core TF modules have been identified in RMS samples: (1) a normal- and tissue-specific module including NR4A1 and MEF2D; (2) a RMS-general module including MYOD1 and MYOG; (3) a fusion-negative (FN)-RMS module including PAX7 and AP-1 members; and (4) a FP-RMS-specific module including MYCN and the oncofusion TFs [34]. In FN-RMS with RAS mutations, aberrant MAPK activity enhanced cell proliferation, whereas blocked myogenic differentiation [35]. Targeted inhibition of RAS pathway with MEK inhibitor (trametinib) rewired substantially the core TF connectivity (modeled by “Coltron”) via MYOG-dependent chromatin remodeling and SE formation at genes required for late myogenic differentiation [35]. Although more biological verification is required to support the subtype-specific CRC in FN-RMS, this study implicates a mechanism that signaling driver mutations integrate with core TF circuitry to promote cancer development.

By far, FP-RMS represents an insightful example of CRC driven by oncogenic fusion TF. Gryder et al. found that P3F reprogramed the cis-regulatory landscape of RMS and established a myogenic SE circuitry together with additional master TFs (MYOG, MYOD, and MYCN) [29]. HiChIP analysis of H3K27ac verified the interactions between core TF genes and their SEs [34]. These core TFs were overexpressed in FP-RMS compared to normal tissues, and collaborated on a myogenic transcriptional program. Ablation of MYOG, MYOD, or MYCN attenuated the expression of all core TFs, while silencing of P3F reduced MYOD and MYCN, but not MYOG. In addition, P3F, MYOG, and MYOD enhanced expression of SOX8, which was a marker for muscle satellite cells and a repressor of transcriptional program associated with differentiation from myoblasts to myotubes [34]. SOX8 also co-localized with other core TFs across the majority of SEs, coordinating the transcriptional chromatin topologies in FP-RMS [67]. The SEs controlling P3F (at FOXO1 locus), MYOD, MYCN, and SOX8 were co-occupied by all core TFs (Fig. 3e). However, MYOG SE was bound by all core TFs except P3F, which was thought to be consistent with the sequential activation of TFs during normal myogenesis. Disruption of SOX8 paradoxically elevated expression of P3F, MYOD1, and MYOG, indicative of a potential negative feedback to block terminal differentiation in FP-RMS. Hence, SOX8 is more likely a co-regulatory TF, which acts as a downstream target of CRC and provides either positive or negative feedback to distinct subsets of core TFs. Importantly, functional screening of SE-associated TFs using a DNA-binding domain-focused CRISPR knockout library affirmed the oncogenic dependency of FP-RMS on SOX8 and the rest of core TFs [34]. Together, P3F and MYCN calibrate the balance between myogenic and anti-myogenic core oncogenic TF circuitries to maintain dedifferentiated state of FP-RMS cells.

Biochemical studies revealed that P3F functioned as a pioneering factor to open chromatin, and subsequently recruited mediator proteins/co-activators (e.g., MED1, p300, BRD4) to enable enhancer looping and transcriptional activation [29]. Functional interplay between P3F and BRD4 yielded a significant susceptibility of FP-RMS cells to BRD inhibition by JQ1. JQ1 destabilized P3F protein and demonstrated a remarkable, selective suppression of P3F targets including core TFs and SE-driven kinases (e.g., ALK). Interestingly, both p300 and HDAC showed extensive co-occupancy with P3F, core TFs and BRD4 across the “epicenters” within SE regions [34, 67]. A JQ1-like SE selectivity was recapitulated by small molecules targeting histone-acetylation writers (p300/CBP) and erasers (HDAC1/2/3), but not general transcriptional modulators (e.g., triptolide, α-Amanitin, and flavopiridol). Triple inhibition of HDAC1/2/3 with either Entinostat or combination of a HDAC1/2 inhibitor Merck60 and a HDAC3 inhibitor LW3 disrupted profoundly the expression of core TFs in FP-RMS cells. Such a SE selectivity of HDAC inhibitors was achieved through excessive increase in the chromatin accessibility of genomic loci, which were co-loaded with both HDAC and core TFs. In contrast, HDAC binding sites without co-binding of core TFs showed a weaker change. As a result of HDAC inhibition, local concentration of acetylated histones was increased around “epicenters.” Aberrant increase in histone acetylation enhanced H3K27ac spreading toward distal boundaries of H3K27ac domains, especially for shorter TF genes (e.g., SOX8, MYOG, MYOD1, and MYCN). Meanwhile, hyper-acetylation of histones also triggered new chromatin contacts at the expense of endogenous, transcription-supportive SE loops within CTCF-defined domains. For instance, Entinostat induced enhancer spreading and aberrant new interactions across 40-kb gap between two MYOD1 SEs, while it diminished their initial interaction. Furthermore, quantitative ChIP-seq analysis (by ChIP-Rx assay) upon HDAC inhibition revealed a general reduction in occupancy of RNA polymerase II, P3F, SOX8, and MYOD1 at regulatory elements of core TFs. In the meantime, genomic binding of MYOG, p300, HDAC2, and HDAC3 remained stable, whereas loading of YY1, RAD21, and BRD4 was significantly increased. The unloading of RNA polymerase II from chromatin may act through two mechanisms: (1) inhibition of pause release at the global scale and (2) alteration of phase separation at core TFs. Notably, HDAC inhibition rapidly dissolved RNA polymerase II condensates, which have been reported to dynamically touch SEs [36–38], while phase-separated BRD4 resisted chromatin hyper-acetylation. Therefore, P3F-driven CRC in FP-RMS is vulnerable to agents targeting the histone-acetylation axis.

#### Dedifferentiated liposarcoma (DDLPS)

DDLPS represents a high-grade subtype of adipocytic mesenchymal tumors. Enhancer profiling of both established cell lines and primary DDLPS tumors enabled discovery of SE-driven FOSL2, MYC, and RUNX1 as top co-operative core TFs (Fig. 3f) [68]. Genomic occupancy analysis revealed a substantial binding and co-loading of BRD2, BRD3, BRD4, FOSL2, and RUNX proteins across active enhancers, especially SE regions of core TFs. Moreover, FOSL2, MYC, and RUNX1 demonstrated inter-dependent expression in DDLPS cells, which was further supported by their robust co-expression in primary tumor specimens. The prominent function of FOSL2 suggests high activity of AP-1 complex in DDLPS. As genomic studies have identified frequent amplification of another AP-1 member JUN in this disease [69], JUN amplification likely contributes to the DDLPS-specific regulatory circuitry. In search of key downstream targets of CRC, SNAI2 was identified as a leading hit whose expression was elevated in DDLPS tumors and was associated with adverse prognosis. Of note, all of the above-mentioned BET bromodomain proteins, core TFs and SNAI2 were essential for both proliferation and tumorigenicity of DDLPS cells. BET protein degraders (e.g., ARV-825 and dBET6) inhibited preferentially SE-associated genes including core TFs and SNAI2, and exerted stronger anti-DDLPS activities than conventional BET bromodomain inhibitors. Therefore, BET protein degraders are promising modalities to subvert oncogenic dependence on disease-driving CRC.

#### Esophageal cancer

Esophageal cancer ranks globally among the top ten most common cancers. This disease can be sub-grouped into esophageal adenocarcinoma (EAC) and esophageal squamous cell carcinoma (ESCC), two entities showing many contrasting features at epidemiological, clinical, and molecular levels [70]. In line with the significant disparities in transcriptome, cancer genome, DNA methylatome, and chromatin profiles, EAC and ESCC display subtype-specific enhancer topography and master TF connectivity [71, 72].

By analyzing EAC-specific SE engagement and co-expression, Chen et al. identified a clique of four master TFs (i.e., ELF3, KLF5, GATA6, and EHF) forming an interconnected circuitry to dictate EAC transcriptome [71]. Overexpression of these four TFs in EAC cells was mutually dependent on the expression of each other. ChIP-seq analysis of ELF3, KLF5, and GATA6 revealed co-occupancy of these TFs at the SE regions of their own and EHF, except for a weak signal of ELF3 binding at GATA6 enhancers (Fig. 3g). Based on integrative analysis of H3K27ac ChIP-seq and circular chromosome conformation capture sequencing (4C-seq), five constituent enhancers (E1–E5) of ELF3 were identified within SE. Among these ELF3 enhancers, E1 demonstrated a strong EAC-specific enhancer activity and was concurrently bound by ELF3, KLF5, and GATA6. Blocking chromatin accessibility of E1 by CRISPR/dCas9-KRAB was able to decrease expression of ELF3, as well as the rest core TFs. Furthermore, ELF3, KLF5, and GATA6 localized in close proximity to each other across the EAC genome. Genes with their enhancers bound by one or more core TFs showed higher expression than those with no binding. Consistent with substantial involvement of these core TFs in EAC transcriptome, they played positive roles to maintain survival and proliferation of EAC cells. SE-driven LIF, LIFR and HNF4A represented leading candidates for CRC downstream targets which exhibited both disease-specificity and therapeutic venerability [71, 73].

In ESCC cells, TP63, SOX2, and KLF5 established a disease-driving CRC to maintain chromatin accessibility and oncogenic transcription (Fig. 3h) [72]. TP63, SOX2, and KLF5 were reported to interact directly with each other and have overlapping occupancy at the SE elements including those of their own. Knockdown of individual core TF abolished expression of all three TFs and downstream targets (e.g., ALDH3A1 and BRD4), which was accompanied by a marked decrease in chromatin accessibility at target enhancers. Moreover, TP63, SOX2, and KLF5 maintained transcription of enhancer RNAs from various TP63 enhancers (e.g., E2, E7, and E8). Strikingly, genomic deletion of these TP63 enhancer elements was sufficient to abolish the expression of all three core TFs and inhibit colony forming ability of ESCC cells. Interestingly, BRD4 was one of the downstream targets of ESCC-specific CRC, as knockdown of any core TF disrupted both transcription and protein expression of BRD4. BET protein degraders (e.g., ARV-771, MZ1, and ARV-825) and HDAC inhibitors (Romidepsin, Panobinostat and JNJ-26481585) were identified as potent anti-ESCC agents. Single agent treatment of Romidepsin degraded TP63 and abolished the expression of all core TFs in a proteasome-dependent manner. Further, combination of Romidepsin and ARV-771 induced substantial chromatin remodeling in ESCC cells, yielding a strong synergistic anti-ESCC effect both in vitro and in vivo.

Interestingly, KLF5 appears as a common core TFs in both EAC and ESCC, albeit the co-operative master TFs are very different. In addition, KLF5 engaged a mutual crosstalk with collaborating factors GATA4 and GATA6 to maintain oncogenic transcriptional regulatory network in gastric cancer [74]. These studies suggest that rewiring connectivity of master TFs is fundamental to disease/subtype identity.

Of note, amplification of SOX2 and TP63 is common in ESCC [75], representing a genomic driver for ESCC-specific CRC. Genomic amplification and somatic SE duplication of KLF5 have also been reported in squamous cell carcinoma, EAC, and gastric cancer [74, 76, 77]. In addition, CCAT1, a SE-driven long noncoding RNA was reported to mediate the binding affinity of SOX2-TP63 complex on target SE (i.e., enhancer of EGFR) [78]. Engagement of noncoding components in CRC may further extend the connectivity and function of TFs.

### Implementation of circuitry analysis in cancer research

Based on findings from biologically verified CRCs, core TFs are vital for cell identity and transcriptional homeostasis under both physiological and pathological conditions. Many other studies have also implemented CRC analysis in uncovering critical transcriptional programs in various cancer types (Table 1) [27, 30, 79–84]. For instance, by analyzing publically available ChIP-seq data from the ENCODE project [85], Fournier et al. modeled core TF connectivity including ESR1, FOXA1, FOSL2, and JUND in MCF7 breast cancer cells; HNF4A, FOXA1, FOXA2, and CEBPB in HEPG2 liver cancer cells; as well as FOXA1, FOXA2, FOSL2, JUND, and ATF3 in A549 lung carcinoma cells [83]. Depletion of FOXA effectively inhibited the viability and clonogenicity of all three cell lines. With information of active chromatin architecture, computational reconstruction of enhancer/NFR-centric CRCs represents an insightful approach to discover TFs with principal functions in a disease/development/subtype-specific transcriptional regulatory network. TF circuitry analysis implicated SOX9, RFX2, SOX2, and ZBTB16 as generally essential factors for ependymoma cells [30]. Top-scored CRC of chronic lymphocytic leukemia predicted PAX5, ETV6, and IRF2 as centralized regulators [27]. Other examples of enhancer/NFR-centric CRC network construction include gastrointestinal stromal tumors [82, 86], multiple myeloma [44], and keratinocyte stem cells [46]. In addition, dbCoRC serves as a valuable database to explore H3K27ac/enhancer-centric core circuitries in over 230 samples including cervical adenocarcinoma, chronic myelogenous leukemia, colorectal cancer, diffuse large B-cell lymphoma, gastric cancer, small cell lung cancer, pancreatic cancer, and prostate cancer.

**Table 1:** Summary of reported core transcriptional regulatory circuitries in cancer.

Cancer type	Core transcription factors	Reference
TAL1-driven T-cell acute lymphoblastic leukemia	TAL1, MYB, RUNX1, and GATA3	[21, 49]
Medulloblastoma (WNT subtype)	LEF1, MAF, RUNX2, and EMX2	[26]
Medulloblastoma (SHH subtype)	OTX1, BARHL2, MAFF, and GLI2	[26]
Medulloblastoma (Group 3)	OTX2, NRL, and CRX	[26, 58]
Medulloblastoma (Group 4)	LMX1A, LHX2, and EOMES	[26]
Neuroblastoma (adrenergic)	MYCN, LMO1, PHOX2B, HAND2, GATA3, ISL1, TBX2, and ASCL1	[28, 32, 62, 63]
PAX3-FOXO1-driven rhabdomyosarcoma	PAX3-FOXO1, MYOD, MYCN, MYOG, and SOX8	[29, 34]
Fusion-negative rhabdomyosarcoma	PAX7, JUNB, JUND, SMAD3, etc.	[34]
Dedifferentiated liposarcoma	FOSL2, MYC, and RUNX1	[68]
Esophageal adenocarcinoma	ELF3, KLF5, GATA6, and EHF	[71]
Esophageal squamous cell carcinoma	TP63, SOX2, and KLF5	[72]
Ependymoma	SOX9, RFX2, SOX2, ZBTB16, etc.	[30]
Chronic lymphocytic leukemia	PAX5, ETV6, IRF2, etc.	[27]
Renal cell carcinoma	PAX8 and HNF1B	[79]
Neuroblastoma (mesenchymal)	NOTCH1, NOTCH2, NOTCH3, MAML2, etc.	[80]
Glioblastoma	KLF4, EGR1, NOTCH1, and SOX2	[81]
Gastrointestinal stromal tumor	FOXF1 and ETV1	[82]
Breast cancer (MCF7 cell line)	ESR1, FOXA1, FOSL2, and JUND	[83]
Liver cancer (HEPG2 cell line)	HNF4A, FOXA2, FOXA1, and CEBPB	[83]
Lung cancer (A549 cell line)	FOSL2, FOXA2, FOXA1, JUND, and ATF3	[83]
B-cell precursor acute lymphoblastic leukemia	MEF2D-fusion, SREBF1, FOS, EGR1, and BCL6	[84]

## Conclusions

The above examples demonstrate the successful application of CRC models to address scientific questions related to cell identity, cancer biology, and therapeutic responses, highlighting the prevalence and importance of CRC in human cancers.

Current paradigm of CRC in human cancers involves four principles: (1) enrolment of master TFs showing high expression and substantial chromatin loading, (2) existence of autoregulation and mutual regulation of core TFs based on either ChIP analysis or motif detection among their regulatory elements, (3) evidence of co-expression of core TFs and transcriptional co-dependencies on each other, and (4) essential function of core TFs and downstream targets in maintaining malignant characteristics of cancer cells.

Computational reconstruction, ChIP-seq, and biologic verification represent the main technical route for CRC studies. Computational algorithms are highly dependent on TF motif analysis. As TFs function in concert with additional co-factors and partners, deviation of actual TF occupancy from motif-based binding sites could compromise the power of computational modeling. Update and refinement of TF motif database warrant more accurate and comprehensive prediction of TF connectivity and network of TFs. Additional efforts can include incorporation of either publically available TF ChIP-seq data or TF ChIP-seq signals from cells of similar identity [87]. In supplement to the enhancer/NFR-centric CRC prediction, integrative analysis of genomic profiling, DNA methylome, chromatin compartmentalization, and enhancer-promoter interactions will extend and clarify regulatory loops. Further improvements in detection and assignment of open chromatin regions to target genes (e.g., 4C-seq) will benefit CRC construction. Since cancer cells are vulnerable to genetic inactivation of core TFs, functional genomics data from genome-wide CRISPR or shRNA loss-of-function viability screening will be useful to interrogate functional importance of candidate core TFs. To this end, the Cancer Dependency Map project (https://depmap.org/portal/), which hosts functional genomics data of over 700 cell lines, serves as a valuable resource to identify TF dependency and cancer vulnerabilities.

Remarkably, both germline polymorphisms and genomic abnormalities can lead to either overexpression or de novo fusion of TFs. These genetically activated TFs are further integrated into the aberrant oncogenic transcriptional programs via CRC formation in collaboration with additional core factors. Driver mutations in signaling pathways can also fuel tumor-promoting CRC via activating their downstream executive TFs. Therefore, CRC operates as a nexus of genomic, signaling, and epigenetic dysregulations in oncogenesis (Fig. 1b). In addition, cancer-associated CRCs often hijack a stem/progenitor cell TF network, which prevents transformed cells from terminal differentiation and in turn conveys information of cell-of-origin. Systematic survey of TF aberrations across human cancer genomes will provide valuable insights for cancer-associated CRC; cancers driven by fusion TFs are fertile grounds of CRC study.

Knowledge of the biologic basis of CRC is growing. CRC frequently enrolls master TFs with pioneer factor activity and/or cancer-specific genomic lesion. Core TFs recruit chromatin/histone modifiers, mediators, and cohesin proteins to reshape epigenetic landscape and establish an oncogenic state [83]. Broad chromatin domain and clustered binding of core TFs at “epicenters” concentrate further co-factors and co-regulators with intrinsically disordered regions (e.g., MED1 and BRD4) to form phase-separated condensates. After compartmentalization, mediator droplets are able to enrich transcription apparatus. Therefore, these condensates in turn provide high local concentrations of trans-activating TFs, chromatin regulators, and transcription machineries for robust expression of target genes (Fig. 1c). Liquid–liquid phase separation emerges as a biophysical mechanism to form dynamic, multivalent condensates of TFs, mediators, and RNA polymerase II to activate SE-associated transcription, especially the expression of core TFs [34, 36–38]. However, regulation and structural basis of the functional dynamics and cooperativity among interacting components within condensates await further exploration. Besides, negative feedback mechanisms that calibrate the strength of feed-forward regulation remain poorly explored.

Therapeutic targeting of histone-acetylation axis and transcriptional apparatus represents an auspicious strategy to disrupt cancer-associated CRC (Table 2). Since BET proteins, CDK7, CDK9, and HDACs, serve as actionable targets in many cancers addicted to CRC, combination of CRC-disrupting agents with either current treatment modalities or novel antitumor compounds may improve therapeutic response. Of interest to note, condensates of RNA polymerase II are sensitive to transcriptional stress, while those of BRD4 and mediators are more resilient [34, 36]. Selective blockage of phase separation or co-inhibition of multiple components in phase-separated condensates may have synergistic effects to break transcriptional circuitry in tumor cells. Although many aspects remain unexplored, plasticity and heterogeneity of CRC have been implicated in human cancers [28, 35]. CRC-guided tumor stratification and therapeutic development are promising fields for future investigation.

**Table 2:** Summary of small molecules targeting the disease-specific core regulatory circuitries.

Molecule		Disease	Antitumor activity	Effect on CRC	Reference	Investigational compound with similar activity	
						Name and function	Clinical status
THZ1	CDK7 inhibitor	T-ALL	In vitro In vivo	Inhibit RUNX1, TAL1, and GATA3	[54]	N.A.	N.A.
SNS-032	CDK9 inhibitor	T-ALL	In vitro	Inhibit RUNX1 and MYB	[55]	AZD4573 CDK9 inhibitor	Phase 1 (NCT03263637) including ALL
NVP2	CDK9 inhibitor	T-ALL	In vitro	Inhibit RUNX1 and MYB	[55]	SCH 727965 CDK1/2/5/9 inhibitor	Phase 2 (NCT00798213) including ALL
THAL-SNS-032	CDK9 degrader	T-ALL	In vitro	Inhibit RUNX1 and MYB	[55]	N.A.	N.A.
JQ1	BET protein inhibitor	T-ALL	In vitro In vivo	Inhibit RUNX1	[56]	OTX015/MK-8628 BET protein inhibitor	Phase 1 (NCT01713582) including ALL
JQ1	BET protein inhibitor	Fusion-positive RMS	In vitro In vivo	Inhibit SOX8, MYOD1, MYOG, and MYCN	[29]	N.A.	N.A.
JQ1 + THZ1	BET protein inhibitor + CDK7 inhibitor	Neuroblastoma	In vitro In vivo	Inhibit MYCN, HAND2, ISL1, PHOX2B, GATA3, TBX2, and ASCL1	[32, 62]	BMS-986158 BET protein inhibitor	Phase 1 (NCT03936465) including childhood cancer
						GSK525762 BET protein inhibitor	Phase 1 (NCT01587703) including neuroblastoma
						SY-5609 CDK7 inhibitor	Phase 1 (NCT04247126) advanced solid tumor^a^
						CT7001 CDK7 inhibitor	Phase 1 (NCT03363893) advanced solid tumor^a^
dBET6	BET protein degrader	T-ALL	In vitro In vivo	Inhibit RUNX1 and MYB	[56]	N.A.	N.A.
dBET6	BET protein degrader	DDLPS	In vitro	Inhibit FOSL2, MYC, and RUNX1	[68]	N.A.	N.A.
Trametinib	MEK inhibitor	RAS-mutated RMS	In vitro In vivo	Rewire core TF connectivity	[35]	N.A.	N.A.
Entinostat	HDAC1/2/3 inhibitor	Fusion-positive RMS	In vitro	Inhibit SOX8, MYOD1, MYOG, and MYCN	[34]	Vorinostat Pan-HDAC inhibitor	Phase 1 (NCT04308330) including RMS
Merck60 + LW3	HDAC1/2 inhibitor + HDAC3 inhibitor	Fusion-positive RMS	In vitro	Inhibit SOX8, MYOD1, MYOG, and MYCN	[34, 67]	Mocetinostat HDAC1/2/3/11 inhibitor	Phase 1 (NCT04299113) in RMS
ARV-825	BET protein degrader	DDLPS	In vitro In vivo	Inhibit FOSL2, MYC, and RUNX1	[68]	N.A.	N.A.
Romidepsin	HDAC inhibitor	ESCC	In vitro In vivo	Inhibit TP63, SOX2, and KLF5	[72]	Vorinostat Pan-HDAC inhibitor	Phase 1 (NCT00537121) including esophageal cancer

*T-ALL* T-cell acute lymphoblastic leukemia, *ALL* acute lymphoblastic leukemia, *RMS* rhabdomyosarcoma, *DDLPS* dedifferentiated liposarcoma, *ESCC* esophageal squamous cell carcinoma, *N.A*. not applicable.

^a^Not specified for the treatment of neuroblastoma.
